# Hope May Come From Internet in Times of COVID-19: Building an Online Programme for Grief (LIVIA)

**DOI:** 10.3389/fpsyt.2021.626831

**Published:** 2021-03-09

**Authors:** Laurent Berthoud, Liliane Efinger, Maya Kheyar, Valentino Pomini, Anik Debrot

**Affiliations:** Institute of Psychology, Faculty of Social and Political Sciences, University of Lausanne, Lausanne, Switzerland

**Keywords:** internet-based interventions for mental health, coronavirus—COVID-19, interpersonal loss, grief, mourning (bereavement), marital separation/divorce

## Covid-19: A Breeding Ground for a Lack of Connection in Times of Grief

As we write these lines, the coronavirus-19 (COVID-19) pandemic has reportedly killed over 2'400'000 people, leaving many individuals and families in mourning throughout the world. The current context has put a major strain on people as it has drastically altered our daily lives and caused many societal challenges. We are experiencing much change and multiple losses. In addition to increased unemployment and financial difficulties, COVID-19 has required exceptional sanitary measures such as social distancing, confinement and quarantine, adding a painful sense of isolation to individuals and families in mourning (1). Simultaneously, this context has had a serious impact on couples, many countries reporting a significant increase in separations and divorces due to spending more time together confined and separating as a result of the exacerbation of pre-existing contextual vulnerabilities that were previously milder or latent (2), adding further grief to already distressing circumstances. Isolation and lack of connection are at the center of these life-changing events. They likely make the grieving and separation processes more complicated and difficult for people who are left alone, without the usual opportunities for interpersonal and social support.

Interpersonal loss, by death or separation, is common, but counts among the most stressful and painful life events possible (3). Both involve the loss of a meaningful relationship and may have significant health consequences, such as enhancing mortality risk and fostering mental or physical illnesses (4–6). Accumulating evidence indicates that interpersonal loss in divorce and breakups has numerous similarities with the grieving process after the death of a loved one. In both cases, symptoms of grief may occur, such as intrusive thoughts, ruminations, avoidance of situations or places reminiscent of the lost person, excessive idealization of the ended relationship, significant fatigue, some mental confusion coupled with the feeling of being lost, etc. (7–9).

Given the current circumstances that this could lead in the next few months to a potential explosion of cases of prolonged grief, it may prove relevant to provide easier access to preventive or even therapeutic psychological interventions for bereaved or separated individuals who are struggling with complicated grief symptoms and who feel the need or are seeking help to overcome their difficulties.

## Filling the “Treatment Gap”: How Internet-Based Interventions can Help

The majority of bereaved people rely on family and friends for support (10, 11) and do not seek professional sources of help. Studies have shown that most individuals in need of mental health services will receive no treatment [also known as the “treatment gap,” see (12)]. Indeed, professional sources are the least used, due to a reported lack of information (e.g., “I've never heard of them”) and availability (e.g., “They're always too busy”). Moreover, professional help is also perceived as highly unhelpful [46% of the respondents found psychiatrists unhelpful, 21% for psychologists; (13)] primarily because of a lack of sensitivity (e.g., “I was told to go sit in the sun and pat the dog”). This highlights the need to improve the dissemination of information and to increase the availability of skilled professionals. Indeed, despite the high probability of experiencing a significant loss in life and the important number of people affected by complicated grief, many professionals do not possess sufficient training and competences (14).

Internet-based interventions (IBIs) represent a promising avenue to address the treatment gap. They are immediately accessible and can reach a large number of individuals. They also diversify the ways to deliver evidence-based treatments (15, 16). IBIs have been shown to be as effective as face-to-face therapies when done with guidance (17). These IBIs generally offer regular but limited personalized support from therapists who guide the patients through the intervention, by email or telephone or video, and rely on psychoeducation and CBT techniques. Guided IBIs, i.e., interventions that offer personalized guidance, are generally more effective than unguided ones [for a systematic review, see (18)]. Nevertheless, guided IBIs require significant human resources, which limits the implementation of the intervention on a large scale.

Although IBIs have only recently started focusing on grief-related symptoms, they have shown promising and stable results, demonstrating their feasibility and efficacy. A recent systematic review and meta-analysis (19) identified 7 RCTs (*N* = 1,257) on guided IBIs, all based on CBT, out of over 4,100 studies. Results showed a promising overall effect on grief reduction with significant moderate effects sizes (Hedge's *g* = 0.54; 95% CI: 0.30–0.78), stable over time from post to 3-month follow-up assessment. To the best of our knowledge, two IBIs targeting grief-related symptoms have been tested to date in an unguided format. In the first study, Dominick et al. (20) proposed an unguided intervention based on psychoeducation. The main goal was to normalize the grief reaction. Their intervention showed positive and significant results, but of small magnitude. In the second study, van der Houwen et al. (21) assessed an unguided intervention, in which participants were asked to complete written disclosure tasks. This 7-week intervention showed positive results on emotional loneliness, rumination and positive mood, however not on grief and depressive symptoms. Both studies showed fewer positive results than those obtained with the guided IBIs.

While most of the interventions were developed for people who were bereaved or suffering from PTSD, one study extented this treatment to other types of loss. Indeed, Brodbeck et al. (22) have developed a 10-week guided IBI, named LIVIA, to treat grief-related symptoms for people who lost their partner either by death or by separation/divorce. This program is based on CBT procedures and emphasizes both loss-focused interventions (e.g., exposure, cognitive reframing of the loss, etc.) and restoration-oriented tasks (e.g., selfcare, social reengagement, etc.) in line with the Dual Process Model of coping with bereavement (23, 24). LIVIA is not only demonstrated to be feasible for both grieving and separated or divorced individuals, but is also efficacious (25).

## The Livia Programme

Few psychotherapists are trained in treating complicated grief (14). Given its prevalence, many people are in need of an intervention in their mother tongue. In 2018, French-speakers represented 5% of the world's population, i.e., 300 million people (26). Nevertheless, no empirically-assessed IBI for grief exists in French. Therefore, we translated the LIVIA programme from German into French. This led to the creation of LIVIA-FR (27), which was evaluated in an unguided format because of limited human resources, in order to test its feasibility in French culture and language. Results from a recent pilot study (28) details that out of 138 interested individuals, 39 participants began the study and 22 were selected for the analyses, 17 having dropped out or not completed a single session. The results showed significant reduction in grief symptoms and a tendency to decrease avoidance strategies. However, smaller effect sizes and higher drop-out rates than the original programme prompted us to develop an upgraded version of the programme, based on the LIVIA-FR participants' feedback and on the literature.

The new version of the programme, named LIVIA 2.0, is currently in development. Like its predecessors, it will consist of 10 sessions to be completed over 3 months. In order to improve the effectiveness of and adherence to the programme, which consists of promoting the autonomy of the participants completing it and reducing the risk of avoidance and drop-out due to feelings of failure, LIVIA 2.0 will include the following changes. First, guidance on demand will be implemented as it is a cost-effective alternative to guidance and will help better meet the participants' needs and expectations with the challenge of making the programme as effective as possible while optimizing the use of human resources (29). No research has been conducted to test the efficacy of a guidance on demand design in participants with complicated grief symptoms. Second, participants will experience greater freedom of navigation so as to choose the order in which they wish to complete the programme according to their needs and abilities. Participants will also receive a personalised recommendation based on the assessment of their priorities at the start of the programme. Third, programme interactivity will be enhanced by displaying a more user-friendly layout, as well as audio files, video files and exercises. This will replace the original textual and academic presentation. Fourth, automated emails will be included in the programme as they are beneficial to adherence and outcomes in IBIs (30). Fifth, the structure of the programme will no longer be linear but modular, addressing cognitions, emotions and behaviours. Sixth, a module addressing autobiographical memory and identity will be added (31) which are central processes that are affected by complicated grief (32, 33). Finally, self-assessment and promotion of the participants' resources will be carried out, using the AERES tool (34).

In the coming years, we have planned to compare the efficacy of LIVIA-FR and LIVIA 2.0. This study is supported by the Swiss National Science Foundation. It is hypothesised that LIVIA 2.0 will require less guidance than LIVIA-FR and be at least as efficient. A more refined exploration will be done on the short-term efficacy of each module by monitoring the participants' state throughout the programme. We also hope that this study will show that the envisaged improvements will be effective and will improve not only access but also, and above all, adherence to the programme.

## Conclusion

Although grief is a natural response to loss, our social context plays a vital role in how we experience these events. Given the circumstances, there is clearly an urgency to offer support to people mourning. IBIs such as LIVIA are promising to meet needs that were already present but are not satisfied or exacerbated by the current sanitary crisis. With such uncertainty and insecurity because of COVID-19, having the support of a programme like LIVIA 2.0 can be “the lifebelt” that can help navigate these turbulent times. Indeed, the current pandemic context has made the grieving process harder. Isolation, social distancing and confinement all have significant effects as we feel as they rob us of relationships crucial to our well-being. The lack of relationships may lead to difficulties in coping with the fear of the unknown in an ambiguous crisis situation as COVID-19. Faced with loneliness, nothing can replace true human contact, but internet-based interventions may serve as an intermediary to build new relationships that may help to overcome mourning. Nevertheless, progress must be made not only in technology but also in the design of programmes to better target needs and offer relevant help to the greatest number. Traditional psychoeducational programmes are perhaps still too standardised and uniform today to respond to the variety of suffering and research has the potential to help guide technology in the right direction. And hopefully, we will be better equipped to support ourselves in times of loss as a result of this pandemic.

## Author Contributions

LB and AD conceived the work. LB, AD, and LE made the literature search. LB drafted the paper. MK, LE, and VP revised the work. All authors provided approval of the version to be submitted.

## Conflict of Interest

The authors declare that the research was conducted in the absence of any commercial or financial relationships that could be construed as a potential conflict of interest.
